# Genomic characterization and phage cocktail suppression of a multidrug-resistant *Vibrio sinaloensis* isolate from diseased shrimp

**DOI:** 10.1128/aem.00680-26

**Published:** 2026-08-20

**Authors:** Zhongfeixue Wang, Jiulong Zhao, Chengcheng Li, Yufei Yue, Zengmeng Wang, Linkai Ou, Yantao Liang, Min Wang, Yongyu Zhang

**Affiliations:** 1 College of Marine Life Sciences, Institute of Evolution and Marine Biodiversity, MoE Laboratory of Evolution and Marine Biodiversity, Frontiers Science Center for Deep Ocean Multispheres and Earth System, Center for Ocean Carbon Neutrality, Ocean University of China 12591, Qingdao, China; 2 Qingdao New Energy Shandong Laboratory, Key Laboratory of Biofuels, Shandong Provincial Key Laboratory of Energy Genetics, Qingdao Institute of Bioenergy and Bioprocess Technology, Chinese Academy of Sciences, Qingdao, China; 3 University of Chinese Academy of Sciences 74519, Beijing, China; 4 Haide College, Ocean University of China 12591, Qingdao, China; 5 Southern Marine Science and Engineering Guangdong Laboratory (Zhuhai) 590852 , Zhuhai, China; Universidad de los Andes, Bogotá, Colombia

**Keywords:** *Vibrio sinaloensis*, putative virulence-associated genes, phage cocktail, phage biocontrol, aquaculture

## Abstract

*Vibrio sinaloensis* has been reported in association with disease events in aquaculture, yet its genomic features and non-antibiotic suppression strategies remain largely unexplored. Here, we report genomic characterization of a multidrug-resistant *V. sinaloensis* strain, ZZ006, isolated from diseased shrimp and evaluate phage-mediated suppression of this strain *in vitro*. The ZZ006 genome harbors multiple putative virulence-associated secretion systems and antimicrobial resistance genes, suggesting chromosomal carriage of traits potentially relevant to host association and antimicrobial tolerance. Using this isolate and its phage-resistant derivatives as hosts, we isolated and characterized three novel bacteriophages (VS1, VS2, VS3) with complementary infection strategies. Their combined application efficiently suppressed ZZ006 growth *in vitro* and reduced the emergence of phage-resistant populations compared with individual phages. Whole-genome resequencing of cocktail-resistant isolates revealed recurrent mutations in pilus- and pseudopilin-associated genes, consistent with receptor-level phage resistance. This study provides genomic characterization of an aquaculture-associated *V. sinaloensis* isolate and establishes an *in vitro* framework for phage cocktail suppression and resistance-evolution analysis, supporting future evaluation of phage-based biocontrol in aquaculture-relevant settings.

**IMPORTANCE:**

*Vibrio sinaloensis* has been detected in diseased aquaculture animals, and multidrug-resistant isolates may complicate disease management. By integrating genomic analysis with experimental phage therapy, this work characterizes a multidrug-resistant *V. sinaloensis* isolate from diseased shrimp and evaluates *in vitro* phage-mediated suppression of this isolate and its phage-resistance evolution. These findings offer actionable solutions for mitigating antibiotic resistance and enhancing sustainability in aquaculture systems.

## INTRODUCTION

*Vibrio sinaloensis* has recently emerged as a significant pathogenic threat in global aquaculture, causing severe disease outbreaks and economic losses in commercially important species such as the Pacific white shrimp (*Litopenaeus vannamei*) and the orange-spotted grouper (*Epinephelus coioides*) (1, 2). Infected shrimp typically exhibit filamentous substances on the pleopods, soft and deteriorated cephalothorax tissue, murky or whitish muscle, and an empty gut, while infected fish show pronounced histological lesions in the liver, kidney, and spleen (1, 2). Outbreaks have been reported across major aquaculture regions in Asia and America (1–5), underscoring the growing global threat posed by this emerging *Vibrio* species.

Despite its increasing prevalence, the virulence mechanisms of *V. sinaloensis* remain poorly understood. Only limited studies have investigated its virulence regulation, with quorum sensing identified as one possible virulence induction pathway (6–8). To date, only one genomic study has been reported, providing a basic genome description without analysis of virulence determinants (7). This limited understanding stands in stark contrast to the extensive genomic characterization of other *Vibrio* pathogens. For instance, *Vibrio parahaemolyticus* harbors multiple virulence genes associated with hemolysin and type III secretion systems (9), while genomic analysis of *Vibrio fluvialis* has linked hemolytic, enterotoxic, and vacuolating activities to its pathogenicity in shrimp (10). Likewise, comparative genomic studies across diverse *Vibrio* species have successfully correlated specific virulence genes, including those encoding metalloproteases, adhesins, and secretion systems, with pathogenicity toward oyster larvae (11–13). A comprehensive genomic insight is critically needed to elucidate the molecular basis of *V. sinaloensis* virulence and to inform effective control strategies.

In addition to its poorly characterized virulence determinants, *V. sinaloensis* poses a serious therapeutic challenge due to its resistance to both antibiotic and immunostimulant treatments (14), leaving conventional disease management strategies largely ineffective. This reflects a broader crisis in aquaculture health management, where excessive antibiotic use has accelerated the emergence of multidrug-resistant pathogens, threatening not only aquaculture species but also environmental and human health (15, 16). Consequently, there is a growing demand for sustainable, antibiotic-free interventions. Among these, bacteriophage (phage) therapy has re-emerged as a promising biocontrol strategy against a variety of aquaculture pathogens including *Vibrio* (17–19), *Shewanella* (19), and *Pseudomonas* species (20).

However, a major limitation of phage therapy lies in the rapid evolution of phage-resistant bacterial variants, which can severely compromise treatment efficacy (21). To address this challenge, we previously developed a combinatorial phage cocktail approach employing phages with complementary infection mechanisms. This strategy achieved sustained suppression of *Vibrio alginolyticus* while minimizing resistance evolution (21), offering a promising foundation for broader phage-based interventions in aquaculture.

In this study, we isolated *V. sinaloensis* strain ZZ006 from diseased shrimp and performed genome-resolved characterization of its antimicrobial resistance and putative virulence determinants. To counteract this strain, we further isolated and characterized three novel phages (VS1, VS2, and VS3) capable of sequential infection against both wild-type and phage-resistant variants. By formulating a synergistic phage cocktail, we achieved efficient suppression of bacterial growth and limited the emergence of phage-resistant mutants. This work not only provides the first genome-resolved insights into the putative virulence and multidrug resistance of *V. sinaloensis* but also establishes a rational phage cocktail as a potential viable and sustainable biocontrol strategy against this multidrug-resistant vibrio.

## RESULTS

### Genomic characterizations of *Vibrio sinaloensis* ZZ006

**Fig 1:**
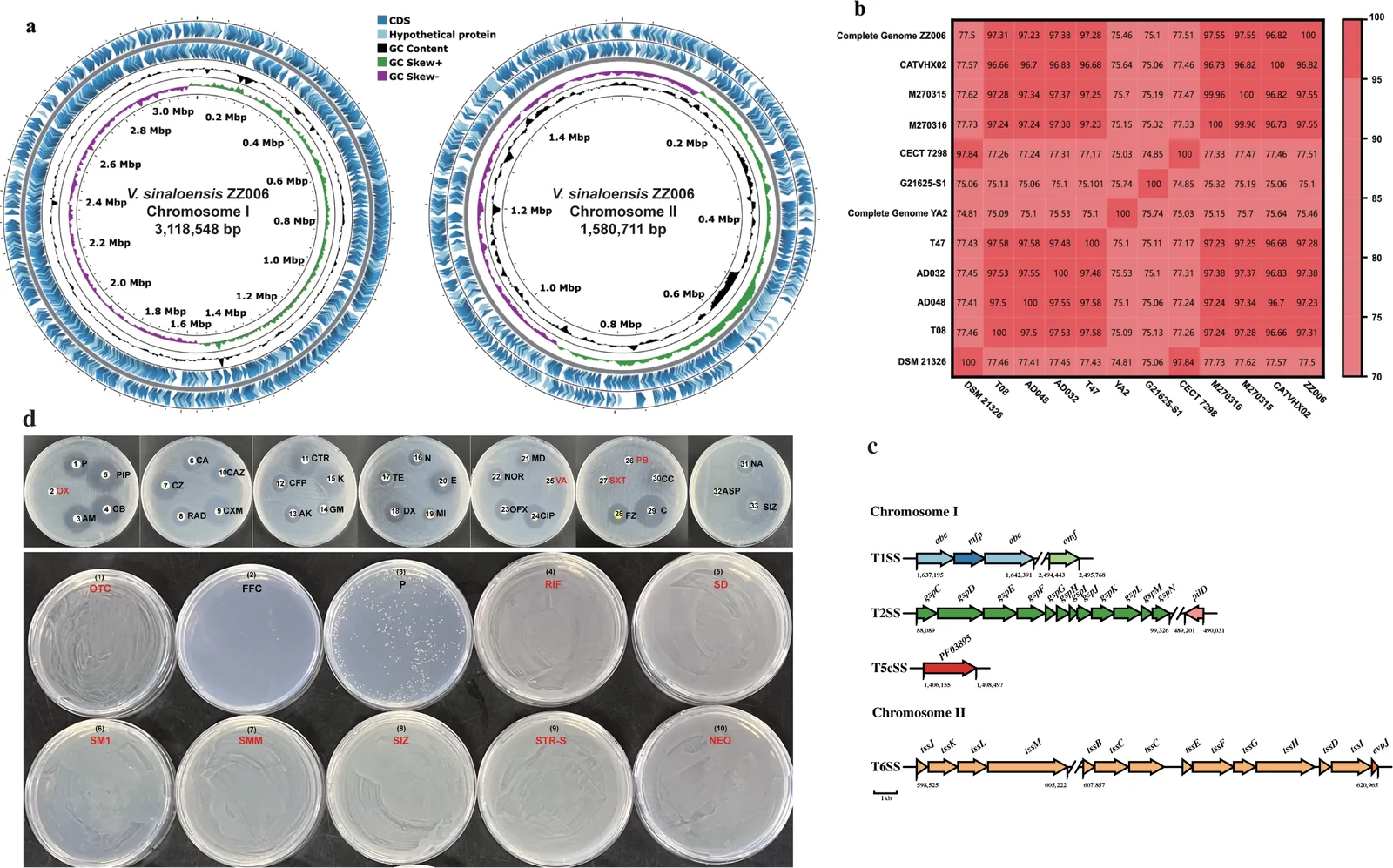
*Vibrio sinaloensis* ZZ006 genome features and antimicrobial susceptibility. (**a**) Circular representation of the complete genome of *V. sinaloensis* ZZ006. The left and right circles correspond to chromosome I and II, respectively. The black ring shows GC content, with positive and negative GC skew indicated in green and purple. The outermost blue ring denotes coding sequences (CDS). (**b**) Average nucleotide identity (ANI) among *V. sinaloensis* ZZ006 and 11 *V. sinaloensis* strains from NCBI (DSM 21326, T08, AD048, AD032, T47, YA2, G21625-S1, CECT 7298, M270316, M270315, and CATVHX02). The heatmap color ranges from pink (moderate identity) to red (highest identity). (**c**) Gene organization of four nearly complete secretion systems. Arrow direction indicates transcriptional orientation, and arrow length is proportional to gene size. Genes of the *gsp* and *tss* families are marked in green and orange, respectively. (**d**) Antibiotic susceptibility of *V. sinaloensis* ZZ006 determined by the standard Kirby-Bauer test (top row) and selective media (bottom panels). Antibiotic abbreviations are labeled on each plate. Red indicates resistance, while black indicates sensitivity. Detailed results are provided in Table S8.

*Vibrio sinaloensis* ZZ006 was isolated from the intestinal tract of diseased *Penaeus vannamei* from a commercial facility located in Hainan province, China, in May 2022. The infected shrimp exhibited typical pathological symptoms of *V. sinaloensis* infection, including filamentous substances on pleopods and soft, deteriorated cephalothorax tissue. Following cultivation in Marine Broth 2216 at 28°C, genomic DNA was extracted and subjected to high-throughput sequencing, yielding a near-complete genome assembly (99.74% completeness, 0.71% contamination) consisting of two chromosomes: chromosome I (3,118,548 bp, 46.21% GC content) and chromosome II (1,580,711 bp, 46.00% GC content) (Fig. 1a). Average nucleotide identity (ANI) analysis with 11 available *V. sinaloensis* genomes in the NCBI database identified 6 strains showing >96% ANI similarity to ZZ006 though none had complete genome sequences (Fig. 1b). The 16S rRNA gene sequence exhibited 99.92% identity with *V. sinaloensis* strain T47 (GenBank accession KM007162.1), confirming species-level identification. Gene annotation identified 2,763 and 1,436 open reading frames (ORFs) on chromosomes I and II, respectively. Functional annotation against the KEGG database characterized 85.0% (2,348/2,763) of chromosome I ORFs and 76.5% (1,099/1,436) of chromosome II ORFs.

Comparison with the Virulence Factor Database (VFDB, September 2025 release) (22) identified 152 putative virulence genes, 141 on chromosome I and 11 on chromosome II (Table S1). These were mainly associated with motility (*n* = 61), adherence (*n* = 26), immune modulation (*n* = 26), and effector delivery systems (*n* = 16) (Table S2). Notably, multiple secretion systems were detected, including type II (T2SS), type IV (T4SS), and type VI (T6SS), which are recognized as canonical virulence determinants in bacterial pathogenesis (23–25). Comprehensive analysis with MacSyFinder (26) further revealed four nearly complete secretion systems: type I (T1SS, 100% completeness), T2SS (100%), type V (trimeric autotransporter, T5cSS, 100%), and T6SS (92.9% completeness) (Table S3; Fig. 1c). Essential genes like *omf* for T1SS, *gspC* for T2SS, PF03895 for T5cSS, and *tssD* for T6SS were identified (27) (Table S3). Genes encoding structural components for motility and adherence apparatus, including the type IV pilus (94.1%), mannose-sensitive hemagglutinin (MSHA) pilus (94.1%), and flagellum (90.9%), showed high completeness (Table S3). Besides, virulence-associated secretion systems and pilus/flagellar motility genes were predominantly located on chromosome I rather than in genomic islands or chromosome II (Tables S2 to S4). Only T6SS genes were located on chromosome II (Table S3). This distribution pattern suggests that these putative virulence determinants represent core genomic features rather than recently acquired mobile elements, suggesting stable chromosomal carriage of these putative virulence-associated systems in ZZ006 (28).

### Antibiotic resistance profiles

Antibiotic susceptibility testing against 43 antibiotics revealed that *V. sinaloensis* ZZ006 was resistant to 12 antibiotics, including oxacillin (OX), vancomycin (VA), polymyxin B (PB), trimethoprim-sulfamethoxazole (SXT), oxytetracycline (OTC), rifampin (RIF), sulfadiazine (SD), sulfamerazine (SM1), sulfamonomethoxine (SMM), sulfisoxazole (SIZ), streptomycin sulfate (STR-S), and neomycin sulfate (NEO) (Fig. 1d; Table S8). Genomic screening identified a total of 10 antimicrobial resistance genes (ARGs) distributed across both chromosomes (Table S1). Chromosome I harbored five ARGs: *vat* (encoding virginiamycin A acetyltransferase), *aacA* (aminoglycoside 6′-N-acetyltransferase), two copies of *aac2-I* (aminoglycoside 2′-N-acetyltransferase I), and *vgb* (virginiamycin B lyase). The phenotypic resistance to the aminoglycosides STR-S and NEO was consistent with the presence of *aacA* and two *aac2-I* copies on chromosome I. Chromosome II carried *qnr/mcbG* (fluoroquinolone resistance protein), *lnuB/lnuF* (lincosamide nucleotidyltransferases), two copies of *catB* (chloramphenicol O-acetyltransferase type B), and *aphA* (kanamycin kinase) (Table S1). Notably, although phenotypic resistance was not observed for certain antibiotics corresponding to the existing ARGs (e.g., kanamycin, chloramphenicol, amikacin, and virginiamycin), their presence suggests ZZ006 may harbor latent resistance potential that could be induced under specific environmental or physiological conditions.

The coexistence of putative virulence-associated systems and multidrug resistance determinants in ZZ006, together with its isolation from diseased shrimp, motivated evaluation of non-antibiotic suppression approaches, while its pathogenicity remains to be experimentally tested.

### Isolations and biological characterizations of phages infecting *V. sinaloensis* ZZ006

**Fig 2:**
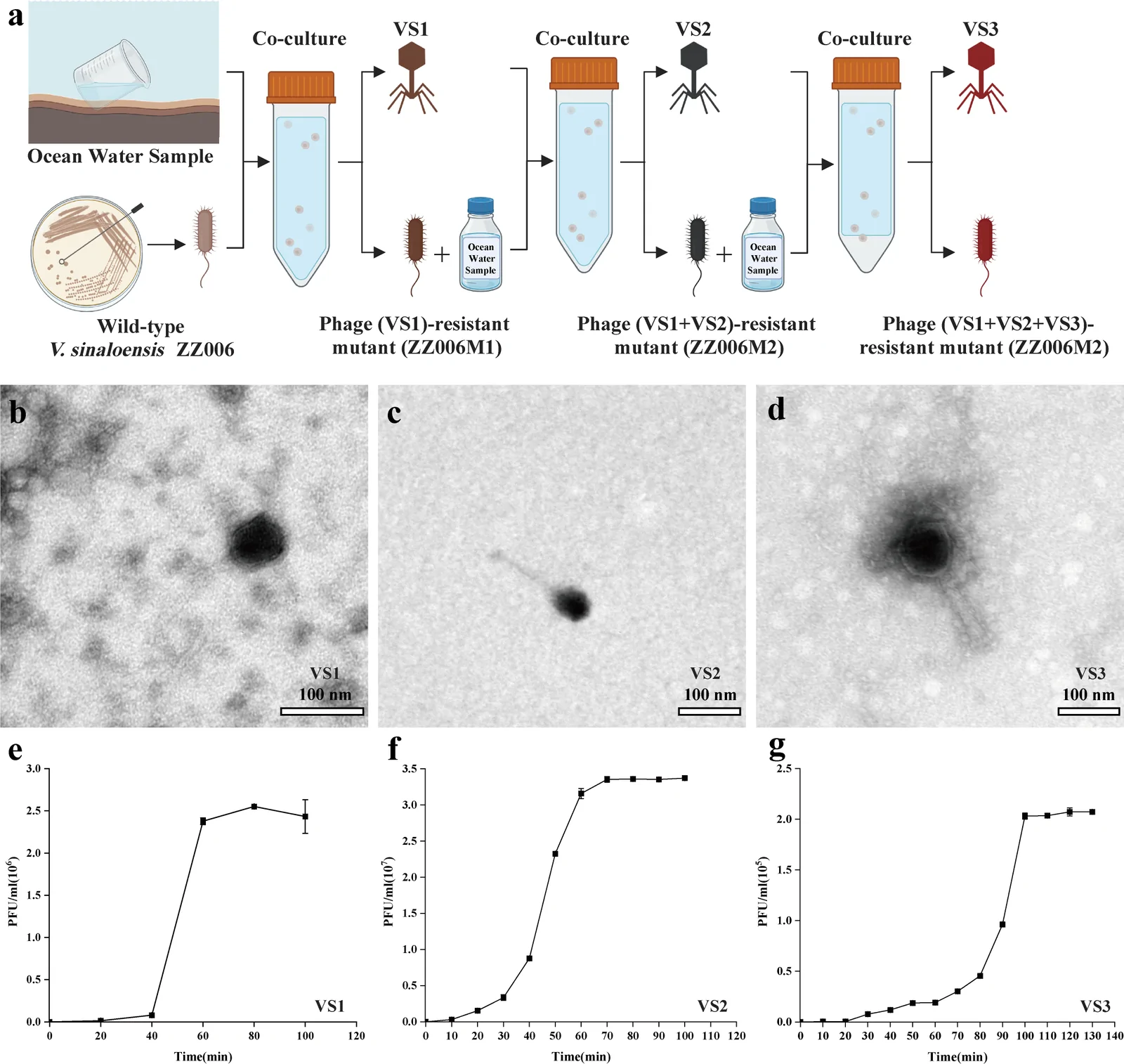
Schematic diagram of the method for isolating phages and phage-resistant mutants (**a**) and morphology (**b–d**) and one-step growth curves (**e–g**) of phages. Transmission electron micrographs of phages show head shape, size, and tail length. One-step growth curves display mean values ± standard error from three biological replicates.

In this study, three lytic phages (VS1, VS2, VS3) were isolated from Yellow Sea coastal waters using wild-type *Vibrio sinaloensis* ZZ006 and its phage-resistant mutants as hosts (21). Specifically, phage VS1 was first isolated using the wild-type *V. sinaloensis* ZZ006 as the host. Phage (VS1)-resistant mutant (ZZ006M1) was then generated by infecting ZZ006 with VS1, and the mutant was used to isolate phage VS2. Finally, ZZ006M2, resistant to both VS1 and VS2, was isolated and employed as the host for isolating phage VS3 (Fig. 2a).

### Phage morphology

Transmission electron microscopy revealed that all three isolates belong to the order *Caudoviricetes* (formerly *Caudovirales*), displaying long contractile tails typical of the *Myoviridae*-like morphology (Fig. 2b through d). Phages VS1 and VS2 possessed icosahedral heads of ~55 nm and ~60 nm diameter, respectively, while VS3 exhibited a markedly larger icosahedral head (~95 nm diameter). Tail fibers measured approximately 111 nm (VS1), 100 nm (VS2), and 130 nm (VS3).

### One-step growth curve

One-step growth curve analysis revealed distinct life cycle characteristics among the three phages when infecting wild-type *V. sinaloensis* ZZ006. The complete lytic cycles lasted approximately 60, 70, and 100 min for VS1, VS2, and VS3, respectively. Latent periods were 20, 10, and 20 min, respectively, but burst sizes varied markedly, with 46 (VS1), 448 (VS2), and 208 (VS3) PFU per cell, respectively (Fig. 2e through g).

### Phage stability

**Fig 3:**
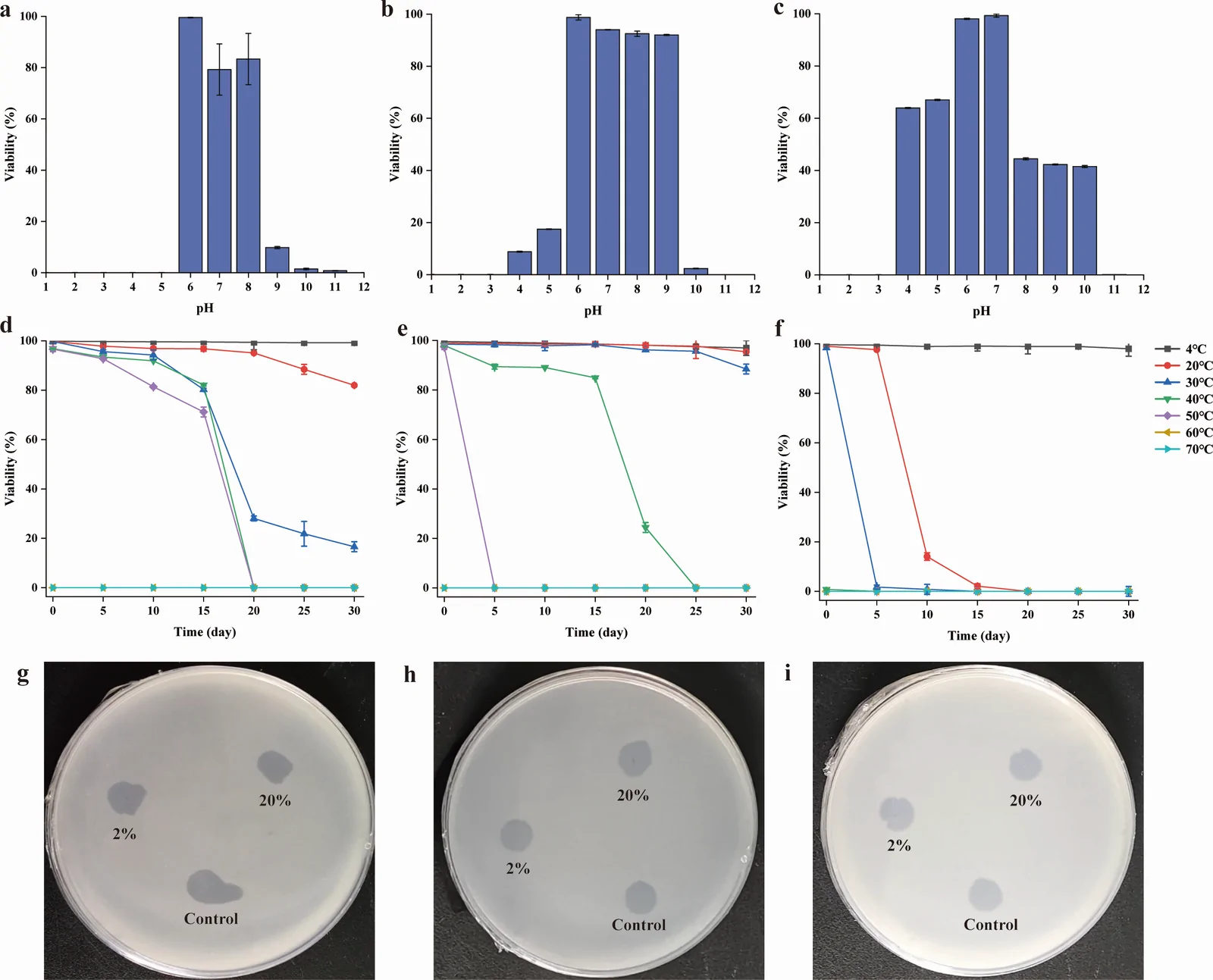
Stability of phages VS1, VS2, and VS3 under different conditions. Phage sensitivity to pH (**a–c**), temperature (**d–f**), and chloroform (**g–i**). In g–i, “Control” denotes untreated samples; “2%” and “20%” indicate treatment with 2% or 20% chloroform.

Phage stability assays revealed distinct tolerance profiles under environmental stress (Fig. 3). Across pH 2–12, VS1 remained stable only under near-neutral conditions (pH 6–8), whereas VS2 showed enhanced stability in weakly alkaline conditions (>90% viability at pH 9), and VS3 tolerated mildly acidic conditions (>60% viability at pH 4). All phages were nearly inactivated at pH ≤3 or ≥11 (Fig. 3). Temperature stability testing (4°C–70°C) revealed that VS2 was the most thermotolerant, maintaining >90% viability at 30°C for 30 days, whereas VS1 and VS3 viability declined to <20% and 0%, respectively. VS2 retained partial viability at 40°C, but all phages were inactivated above 50°C (Fig. 3). None were affected by chloroform, supporting non-enveloped, proteinaceous capsid structures (Fig. 3).

### Genomic and phylogenetic features of phages VS1, VS2, and VS3

Phages VS1, VS2, and VS3 possessed circular double-stranded DNA genomes of 59,962, 59,836, and 205,940 bp, respectively, with GC contents of 46.3%, 45.3%, and 33.1%. Genome annotation identified 79, 78, and 327 ORFs in VS1, VS2, and VS3, respectively, of which 22.8%, 33.3%, and 22.6% could be functionally assigned (Table 1). Identified genes were primarily related to DNA replication (e.g., helicase), DNA metabolism (e.g., endonuclease), DNA packaging (e.g., terminase large/small subunits), and structural proteins (e.g., capsid components) (Tables S5 through S7), including 3,2,2 tail fiber proteins and 0,0,3 baseplate proteins encoded by three phages, respectively (Table 1). The VS1 and VS2 genomes each encoded one tRNA, while VS3 contained the largest tRNA complement (10 total) (Table 1). Importantly, no virulence genes, antibiotic resistance genes, or lysogenic conversion genes (e.g., integrase, excisionase, transposase) were detected in VS1 and VS2 genomes (Tables S5 and S6). However, VS3 harbored three potential virulence factors (two *groELs* and one *htpB*) (Table S7).

**TABLE 1:** Basic genome characteristics of phages VS1, VS2, and VS3

Characteristic	VS1	VS2	VS3
Genome length (bp)	59,962	59,836	205,940
GC content (%)	46.3	45.3	33.1
tRNA	1	1	10
ORFs	79	78	327
Functionally annotated ORFs	18	26	74
Tail fiber protein	3	2	2
Baseplate protein	0	0	3

Comparative genomics revealed limited homology among the three phages. VS1 and VS2 shared ~20% genome coverage (81.0% identity), whereas VS3 showed no detectable similarity to either. Against the NCBI viral database, VS1 and VS2 exhibited low similarity to *Vibrio* phage V05 and *Bacillus* phage Thurquoise, respectively (<95% identity, insufficient for species assignment), while VS3 displayed no significant matches, suggesting it represents a highly novel species (29, 30).

**Fig 4:**
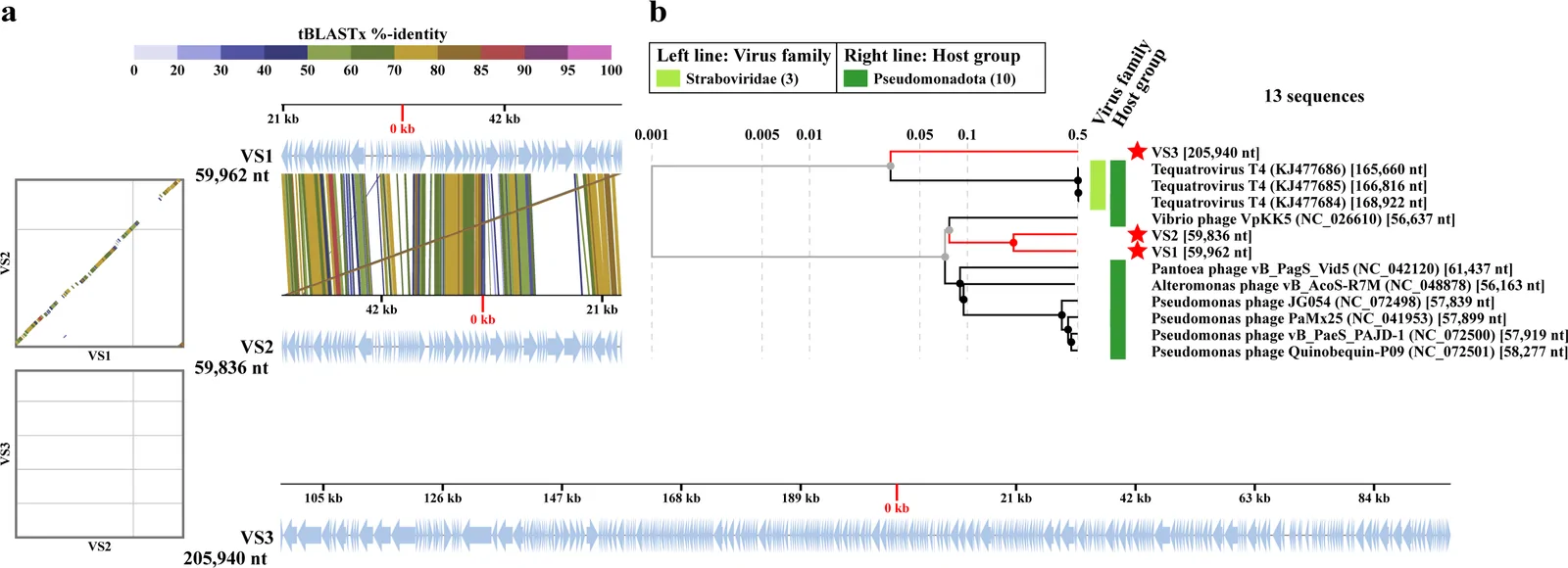
Multiple-genome alignments and proteomic tree of phage VS1, VS2, and VS3. (**a**) Whole-genome alignments of VS1, VS2, and VS3 using tBLASTx. The boxed region highlights homologous genome segments meeting the *E*-value threshold. (**b**) Proteomic tree generated by ViPTree showing VS1, VS2, and VS3 (red stars) and their closest relatives. Ten additional reference phage genomes are included with names, accession numbers, and genome lengths.

Proteomic tree analysis clustered VS1 and VS2 within a monophyletic clade (40.9% SG score), whereas VS3 formed an independent branch with members of the *Kyanoviridae* family, sharing <15% similarity with any known phage (Fig. 4a). Given that >15% similarity defines genus-level clustering, VS3 likely constitutes a new genus within *Kyanoviridae*. Synteny analysis further supported major genomic divergence between VS3 and the other phages (Fig. 4b).

### *In vitro* antibacterial efficacy of individual and combined phages

**Fig 5:**
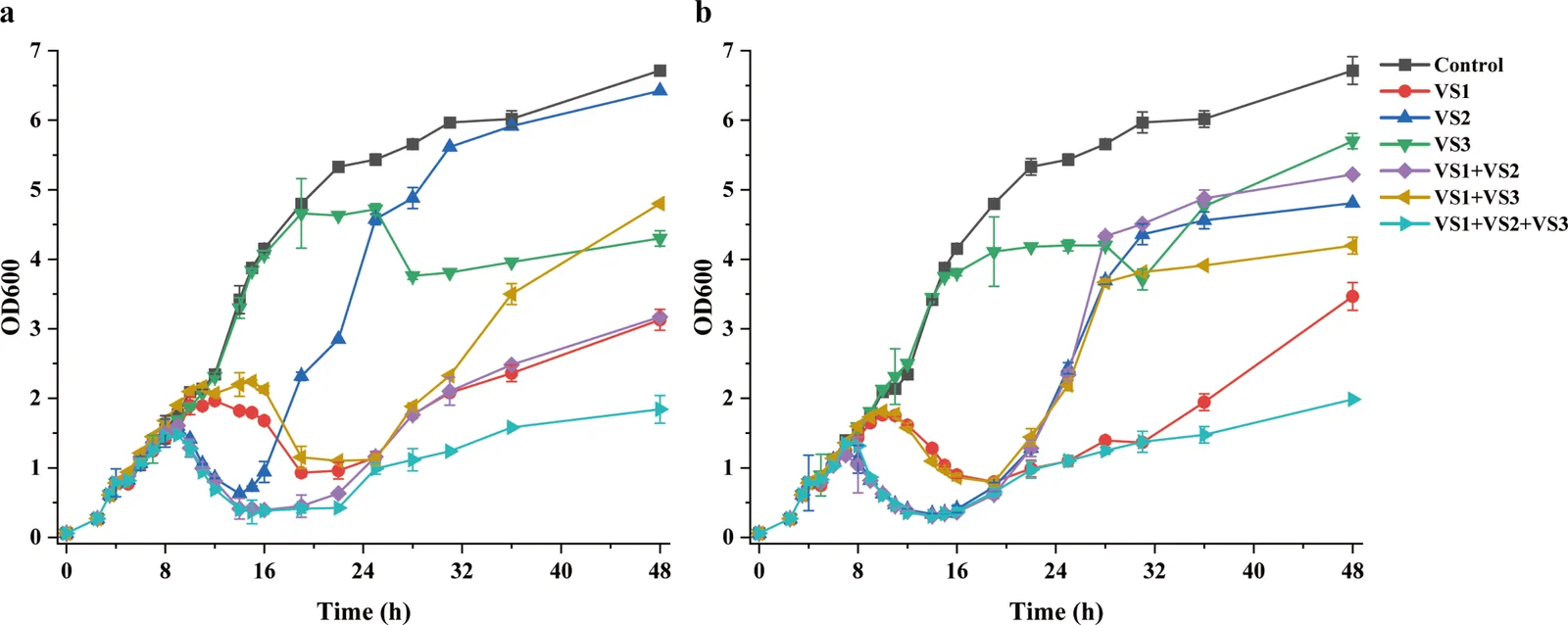
Growth dynamics of *V. sinaloensis* ZZ006 infected by phages VS1, VS2, VS3, and their combinations. Growth curves at MOI 0.1 (**a**) and MOI 1 (**b**). The black line represents the control (ZZ006 only). Red, blue, and green denote infection with VS1, VS2, and VS3, respectively. Purple and brown represent binary combinations (VS1 + VS2 and VS1 + VS3), and cyan indicates the triple combination (VS1 + VS2 + VS3). Error bars represent standard deviations of three independent experiments.

Growth assays of *V. sinaloensis* ZZ006 at MOI = 0.1 revealed distinct phage infection dynamics. VS1 and VS2 suppressed bacterial growth for ~19 and 14 h, respectively, after which optical density (OD_600_) increased, likely due to resistant mutant emergence. VS3 exhibited weaker control, delaying growth only between 19 and 28 h (Fig. 5a).

Two-phage cocktails (VS1 + VS2 or VS1 + VS3) moderately enhanced early suppression but failed to sustain long-term inhibition, with bacterial regrowth observed after ~25 h. In contrast, a three-phage cocktail (VS1 + VS2 + VS3) maintained effective bacterial suppression for >22 h and achieved the lowest final bacterial density (OD_600_ < 2), demonstrating clear synergistic effects. These results indicate that effective phage cocktail design requires sequential isolation of phages targeting successive resistant mutants rather than simple binary combinations (Fig. 5a).

At MOI = 1, higher phage concentrations accelerated bacterial lysis onset but also promoted faster emergence of resistant variants, particularly with VS3 alone or the VS1 + VS2 combination (Fig. 5b). Nevertheless, the three-phage cocktail remained the most sustained bacterial suppression and minimal resistance development (Fig. 5b).

Host range analysis confirmed the broader infection spectrum of the cocktail, which lysed 6 of 26 tested *Vibrio* strains, compared to 3, 4, and 3 for VS1, VS2, and VS3 alone, respectively (Table 2). This expanded lytic range underscores the advantage of rationally designed phage cocktails for suppressing multidrug-resistant *Vibrio* isolates.

**TABLE 2:** Host range of phages VS1, VS2, VS3, and the cocktail^*a*^

Species	Strain	VS1	VS2	VS3	Cocktail
*Vibrio sinaloensis*	ZZ006	+++	+++	+	+++
*Vibrio sinaloensis*	ZZ006M1	−	+++	++	+++
*Vibrio sinaloensis*	ZZ006M2	−	−	+++	+++
*Vibrio campbellii*	18-1	++	−	−	+++
*Shewanella algae*	XJK002	+	+	−	+
*Shewanella upenei*	XJK015	−	+	−	+
*Vibrio parahaemolyticus*	05-1	−	−	−	−
*Vibrio alginolyticus*	VIB 283	−	−	−	−
*Vibrio harveyi*	VIB 645	−	−	−	−
*Vibrio hyugaensis*	24002	−	−	−	−
*Vibrio jasicida*	B2	−	−	−	−
*Vibrio fortis*	HS3-4	−	−	−	−
*Vibrio owensii*	B1	−	−	−	−
*Vibrio rotiferianus*	25006	−	−	−	−
*Vibrio rumoiensis*	JK018	−	−	−	−
*Vibrio mytili*	XJK003	−	−	−	−
*Vibrio xuii*	HNXS002	−	−	−	−
*Vibrio sagamiensis*	XJ001	−	−	−	−
*Vibrio bivalvicida*	HS3-1	−	−	−	−
*Vibrio scophthalmi*	PMP022	−	−	−	−
*Vibrio natriegens*	15-1	−	−	−	−
*Vibrio kanaloae*	HS4-4	−	−	−	−
*Vibrio hepatarius*	HS4-3	−	−	−	−
*Vibrio atlanticus*	E-HMS2016	−	−	−	−
*Vibrio chagasii*	HS4-5	−	−	−	−
*Vibrio pomeroyi*	HS2-2	−	−	−	−

^*a*^ +++ indicates strong lytic activity, ++ indicates moderate lytic activity, + indicates weak lytic activity, and − indicates no lytic activity.

### Pilus assembly-associated mutations contribute to resistance of ZZ006 to the three-phage cocktail

**Fig 6:**
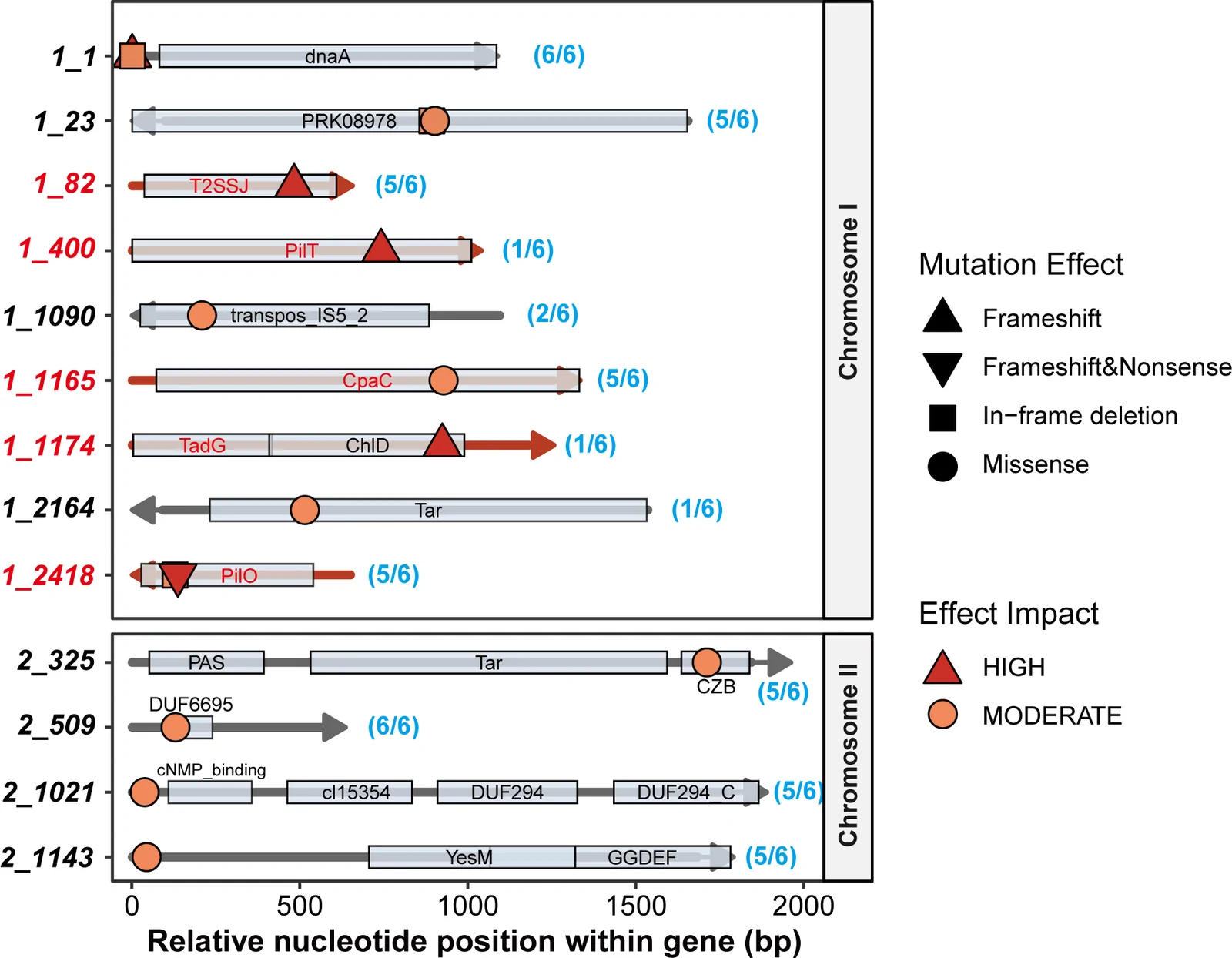
Domain organization and mutation positions of genes carrying high- or moderate-impact mutations in phage cocktail-resistant ZZ006 isolates. Each gene is shown as a directional arrow according to its coding orientation. Conserved domains are indicated by shaded boxes and labeled directly. Mutation sites are marked by symbols, with shape indicating mutation type and fill color indicating predicted impact. The figures in brackets represent the number of resistant isolates carrying the mutation. Pilus assembly-associated genes are highlighted in red.

Although the three-phage cocktail substantially suppressed the emergence of resistant populations in ZZ006 (Fig. 5), resistant mutants (designated ZZ006M3) were still recovered. To elucidate the genetic basis of the evolved resistance, six independent cocktail-resistant isolates were subjected to whole-genome resequencing, and their ability to grow in the presence of the phage cocktail was confirmed. A total of 13 genes carrying high- or moderate-impact mutations (including frameshift, missense, in-frame deletion, and nonsense mutations) were identified across the six isolates (Fig. 6; Table S9). Of these, nine genes were recurrently mutated in five or all six isolates, indicating strong convergent evolution under phage selection (Fig. 6). Notably, two of these recurrently mutated genes (1_82 and 1_1165) were involved in pilus or pseudopilin assembly associated with the type II secretion system, one gene (1_2418) was involved in pilus assembly associated with the type IV secretion system, and the mutations were localized within conserved domains (Fig. 6). In addition to these recurrent mutations, two genes (1_400 and 1_1174) associated with type IV pilus assembly were mutated in only a single isolate (Fig. 6). The occurrence of pilus-related mutations in both recurrent and isolate-specific patterns highlights the potential importance of pilus assembly in mediating resistance to the phage cocktail. All pilus assembly-associated mutations were located on chromosome I (Fig. 6). Given that pili commonly function as receptors for phage adsorption, these mutations likely impair phage attachment, thereby conferring resistance. In contrast, the remaining recurrently mutated genes were associated with nucleotide replication or transfer, acetolactate biosynthesis, and bacterial motility (Fig. 6; Table S9). As these functions are not typically linked to direct phage resistance mechanisms, they may contribute indirectly, potentially through physiological or regulatory changes affecting host-phage interactions.

## DISCUSSION

*Vibrio sinaloensis* has recently emerged as a significant pathogen in aquaculture systems, posing serious challenges to shrimp health and productivity. In this study, we characterized *V. sinaloensis* ZZ006, a multidrug-resistant isolate obtained from diseased *Penaeus vannamei*, and evaluated phage-mediated suppression of this isolate *in vitro*. We emphasize that no shrimp or fish challenge experiment was performed; therefore, ZZ006 should not be regarded as a confirmed etiological agent. However, our results provided necessary information on antimicrobial resistance determinants and putative virulence-associated traits that warrant future functional and infection studies.

Genomic analyses revealed 152 putative virulence-associated genes, including key toxin genes such as *rtxB/C/D/E/H* (MARTX toxin cluster) and *tlh* (thermolabile hemolysin) (31–34), as well as multiple secretion systems (T1SS, T2SS, T5cSS, and T6SS). These systems are recognized as central virulence determinants across both animal and plant pathogens, mediating host colonization and effector translocation (35–38). Their co-occurrence in *V. sinaloensis* ZZ006 hinted a potential infection strategy combining environmental adaptability, host recognition, and cytotoxicity. According to previous studies, this putative virulence-associated gene repertoire (152 genes) falls within the range reported for shrimp- and fish-associated *Vibrio* isolates (39–41), suggesting its pathogenic potential.

Phenotypic and genotypic analyses revealed that *V. sinaloensis* ZZ006 is resistant to at least 12 antibiotics, including oxytetracycline, rifampin, sulfadiazine, and streptomycin sulfate, consistent with the presence of multiple antibiotic resistance genes across both chromosomes. Such multidrug resistance mirrors reports of treatment failures in shrimp aquaculture (42, 43), emphasizing the declining efficacy of conventional antibiotic-based management. Compared with reported aquaculture-associated *Vibrio* genomes, ZZ006 carries a relatively expanded resistance repertoire (10 ARGs), exceeding the average ARG burden reported for 1,036 *V. parahaemolyticus* genomes (*n* = 5 ± 2) (44) and the 1–2 acquired ARGs commonly detected in *V. anguillarum* (45) although it is lower than exceptional multidrug-resistant isolates such as *V. harveyi* 345 (*n* = 25) (46).

The dual presence of putative virulence factors and multidrug resistance (Table S1, Fig. 1d) underscores its potential pathogenicity, and these genomic and phenotypic features provided the isolate-specific context for evaluating phage-mediated suppression of ZZ006.

Phage therapy is re-emerging as a precise and environmentally sustainable solution for bacterial disease control in aquaculture (47–54). Yet, its success is often hindered by the rapid evolution of phage-resistant mutants. Building on our previous work in *Vibrio alginolyticus* (21), we employed a sequential isolation strategy to obtain three novel *V. sinaloensis* phages, VS1, VS2, and VS3, capable of infecting both the wild-type and successive resistant mutants (ZZ006M1, ZZ006M2). Their distinct host ranges (Table 2) suggest they employ different infection strategies against *V. sinaloensis* (21), consistent with observations in *Salmonella* phages that exploit different surface receptors such as O-antigens and outer-core oligosaccharides (55). All three phages demonstrated short latent periods and robust lytic activity, along with high stability across a wide pH range (4–11) and moderate temperatures (30–40°C), demonstrating robustness under aquaculture conditions (56–58). Genomic analysis confirmed the absence of known lysogenic or virulence-associated genes (*recA*, *CI*, *Cro*) in any of the three phages, and no known virulence genes were identified in VS1 or VS2. Although VS3 harbors genes annotated as virulence-related chaperones (*groEL*, *htpB*), these are involved in protein folding rather than toxicity (59, 60), supporting their safety for therapeutic application.

Phylogenetic analysis placed VS1 and VS2 within the subfamily *Queuovirinae*, while VS3 represents a novel lineage within the order *Pantevenvirales* (Fig. 4). Phages of the *Queuovirinae* subfamily are characterized by a suite of traits conducive to therapy, including short latent periods, large burst sizes, environmental robustness, and a relatively narrow host range. Notably, their genomes are devoid of virulence, antibiotic resistance, or lysogenic genes (61–63). In contrast, *Pantevenvirales* phages are distinguished by potent bacteriolytic capabilities, mediated by diverse lytic enzymes embedded within tail fiber and baseplate structures (64–75). For example, the *Pantevenvirales* phage vB_KpnP_W17 encodes capsule-degrading lysozymes that facilitate penetration of bacterial defenses (70). Similarly, against pathogens such as the aquaculture pathogen *Shewanella algae* (19), antibiotic-resistant *Klebsiella pneumoniae* (76), and high-risk avian pathogenic *Escherichia coli* (77), phage cocktails composed of taxonomically diverse phages have also been shown to be effective in suppressing pathogenic bacteria. Thus, combining phylogenetically and mechanistically distinct phages provides a synergistic therapeutic platform to suppress pathogen growth while minimizing phage resistance emergence.

The growth assays of ZZ006 with phages demonstrated that VS3 alone exerted a relatively weaker control over the bacterial growth, delaying growth only after 19 h (Fig. 5a and b). This delayed effect could be attributed to the slower infection kinetics of VS3 compared to other phages (Fig. 2D through F), which may be due to the complex replication and assembly process of this jumbo phage. However, VS3 and its taxonomic relatives, such as phages from the *Pantevenvirales* order, likely possess potent bacteriolytic capabilities. This is supported by the presence of diverse lytic enzymes in VS3, including cell wall hydrolase and tail lysozyme (Table S7), as well as in other *Pantevenvirales* phages (70). We propose that the delayed impact of VS3 on ZZ006 could be due to the gradual accumulation of the phage within the host, with VS3 likely targeting and eliminating or suppressing biofilms after sufficient replication. This hypothesis may also explain why the addition of VS3 enhances the antibacterial efficacy of the two-phage cocktail (VS1 + VS2), potentially through biofilm disruption in addition to its direct infection effects. Together, these features suggest that VS3 employs a more autonomous infection strategy with enhanced adaptability, traits advantageous for durable therapeutic design.

Bacteria deploy diverse anti-phage defenses, including receptor modification, CRISPR-Cas systems, and restriction-modification pathways (78, 79). Consequently, utilizing phages with different infection strategies is key to overcoming these defenses, as they can target different bacterial receptors and circumvent various immune pathways in a complementary manner (80). This approach maximizes the fitness costs of resistance and constrains potential evolutionary escape routes, thereby ensuring the long-term efficacy of phage therapy (81, 82). This study further revealed that effective cocktail design requires sequential isolation of phages against successive resistant mutants, a process essential for achieving durable antibacterial efficacy in dynamic aquaculture environments.

**Fig 7:**
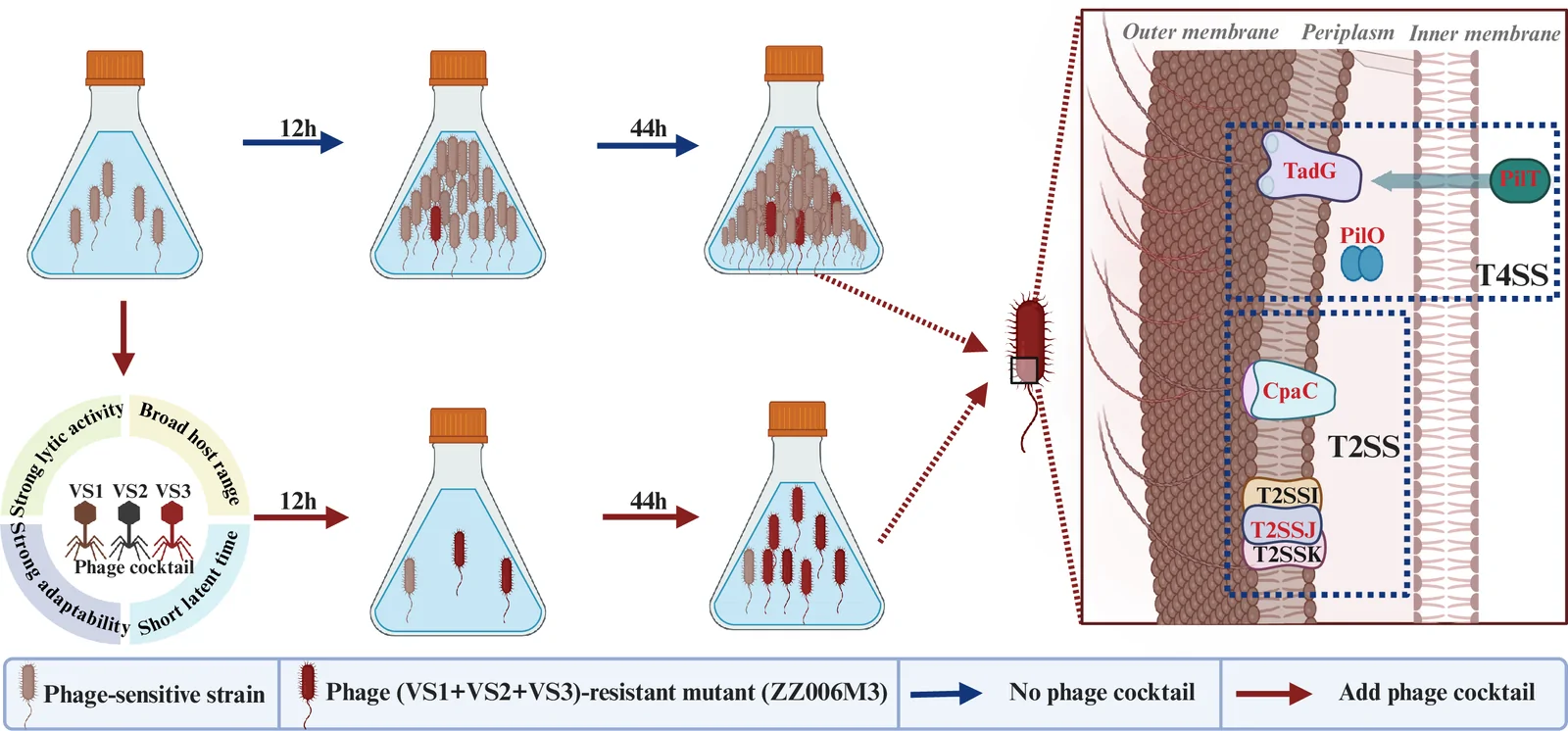
Schematic representation of the responses of the *V. sinaloensis* ZZ006 population to *in vitro* three-phage cocktail treatment. Red arrows indicate bacterial responses under three-phage selection pressure, whereas blue arrows indicate responses in the absence of phage pressure. The enlarged panel on the right highlights proteins encoded by genes carrying mutations (shown in red) that are involved in pilus assembly in phage-resistant mutants. CpaC: Pilus assembly protein (T2SS); T2SSI: Minor pseudopilin (T2SS); T2SSJ: Pseudopilin (T2SS); T2SSK: Minor pseudopilin (T2SS); PilO: Pilus inner membrane component (T4SS); PilT: Pilus retraction ATPase (T4SS); TadG: Pilus assembly protein (T4SS).

We acknowledge that, despite the use of a three-phage cocktail composed of phages with distinct infection strategies, resistant mutants still emerged (Fig. 5 and 7), underscoring the strong adaptive capacity of ZZ006 under phage selection pressure. Whole-genome resequencing revealed that resistance was frequently associated with mutations in genes involved in pilus or pseudopilin assembly, with mutations mainly occurring within conserved functional domains (Fig. 6), suggesting strong selection on structures mediating phage adsorption. This interpretation is consistent with previous studies showing that modification, masking, or loss of cell-surface receptors is one of the most common mechanisms of phage resistance, particularly for receptors such as pilus and flagella (83, 84). In addition, mutations affecting secretion systems have been widely reported to contribute to phage resistance (85), including the evidence that mutations in type II secretion-associated pilus assembly genes mediate resistance in *Ralstonia solanacearum* (86). More broadly, receptor alteration has been widely recognized as an evolutionarily accessible and frequently selected strategy under strong phage pressure (83, 84). In this context, the recurrent emergence of pilus-associated mutations across independent ZZ006M3 isolate supports the conclusion that pilus remodeling represents a major adaptive route to resist the three-phage cocktail. In contrast, mutations in genes related to replication, metabolism, and motility are less likely to directly affect phage adsorption and may instead contribute indirectly by altering cellular physiology or regulatory networks that influence host-phage interactions (84, 87).

From an applied perspective, previous studies have demonstrated that phage cocktails are generally more effective than single phage in delaying resistance, particularly when they target different bacterial receptors (80) or employ distinct infection strategies (21). More recent work has further emphasized the importance of rational cocktail design to constrain resistance evolution (88). However, our results indicate that even a cocktail composed of phages with different infection strategies was insufficient to completely prevent resistance in ZZ006 (Fig. 5 and 7), suggesting that receptor diversification alone may reduce, but not eliminate, resistance emergence. Therefore, future strategies for controlling this pathogen will likely require iterative approaches, including the isolation or evolution of additional phages capable of infecting pilus-deficient or pilus-modified mutants. Such resistance-informed phage selection and cocktail design have been proposed as an effective strategy to improve the robustness of phage therapy (21, 88, 89). Besides, because these antibacterial assays were performed *in vitro*, the efficacy of the three-phage cocktail should be further validated in shrimp challenge experiments, pond microcosms, or other aquaculture-relevant settings. Such studies will be essential to determine ZZ006 pathogenicity, phage persistence, dosing stability, host safety, and potential impacts on resident microbial communities before practical application.

In conclusion, this study provides genome-resolved characterization of a multidrug-resistant *V. sinaloensis* strain isolated from diseased shrimp and demonstrates that a sequentially isolated three-phage cocktail can suppress this strain *in vitro* while selecting for receptor-associated resistance mutations. By connecting isolate-level genomic features with phage susceptibility and resistance evolution within the same ZZ006 genetic background, this work provides a framework for genome-informed phage-host interaction studies and supports future evaluation of phage-based biocontrol in aquaculture-relevant settings.

## MATERIALS AND METHODS

### Bacterial strain isolation

The diseased Pacific white shrimp (*Litopenaeus vannamei*) was obtained from a Hainan farm. The hepatopancreas of the shrimp sample was transferred into a 50 mL sterile centrifuge tube and homogenized using a flame-sterilized glass rod. The mixture was thoroughly mixed with SM Buffer and diluted to 10^−8^. Aliquots (100 µL) from each dilution were spread onto TCBS agar and incubated at 28°C for 8–24 h. Individual colonies were subjected to three rounds of purification by streaking on TCBS plates. Pure isolates were stored at −80°C in 25% (vol/vol) glycerol.

### Bacterial antibiotic resistance testing

Initial screening employed 33 commercial antibiotic discs (Hangzhou Microbial Reagent Co., Hangzhou, China) on 2216E agar plates (90). Bacterial suspensions (OD_600_ = 0.8) were evenly spread using sterile swabs, antibiotic discs were applied, and plates were incubated at 37°C for 24 h. Inhibition zones indicated sensitivity; absence indicated resistance (91).

Expanded testing included aquaculture-relevant antibiotics (92). Diluted bacterial suspensions (100 µL, OD_600_ = 0.8) were plated on selective media containing specific antibiotics at recommended concentrations: oxytetracycline (30 µg/mL), florfenicol (30 µg/mL), penicillin G (50 µg/mL), rifampin (4 µg/mL), sulfadiazine (30 µg/mL), sulfamerazine (30 µg/mL), sulfamonomethoxine (30 µg/mL), sulfisoxazole (16 µg/mL), streptomycin sulfate (30 µg/mL), and neomycin sulfate (8 µg/mL). Plates were incubated at 37°C for 24 h; bacterial growth indicated resistance (93–95).

### Bacterial genome sequencing and analysis

Genomic DNA was extracted from *V. sinaloensis* ZZ006 using the SDS-column purification method. Libraries were prepared and sequenced on the PromethION platform (Oxford Nanopore Technologies) using native barcoding kits (SQK-LSK109 with EXP-NBD104/114) and Illumina HiSeq. Raw reads were quality-filtered using SeqPrep (https://github.com/jstjohn/SeqPrep) and Sickle (https://github.com/najoshi/sickle). Hybrid assembly was performed with Unicycler (v0.4.9) (96), miniasm (0.3-r179) (97), and Racon (v1.4.16) (98), followed by polishing with Pilon v1.23 (99). Assembly quality was assessed using CheckM v1.2.3 (100).

Open reading frames were predicted with Prodigal v2.6.3 (101). Functional annotation utilized KOfamScan v1.3.0 (102) against the KEGG database (103). Virulence factors were identified by BLASTP searches against the VFDB core data set (September 2025 release) using cutoffs of *E*-value <10^−5^, identity >50%, and coverage >50% (104). Protein secretion systems were predicted using MacSyFinder v2.1.5 (105) with TXSScan v1.1.3 models (26), retaining systems with >90% completeness. Genomic islands were identified using IslandViewer 4 (106). Circular genome maps were generated using Proksee (107), and average nucleotide identity was calculated with IPGA v1.09 (108).

### Phage isolation and purification

Phage VS1 was isolated using *V. sinaloensis* ZZ006 as host. Environmental water samples (250 mL each from offshore and farm sources) were filtered (0.22 µm), mixed with equal volumes of log-phase host culture in double-concentrated 2216E broth, and incubated at 28°C with shaking. Samples were collected daily for 7 days, filter-sterilized, and subjected to plaque assays. Phage lysates (100 µL) were mixed with 100 µL host culture, incubated for 20 min at 28°C, combined with 5 mL molten 0.5% soft agar, and overlaid on 1.5% 2216E agar plates. Individual plaques were picked, eluted in SM buffer (50 mM Tris-HCl pH 7.5, 100 mM NaCl, 10 mM MgSO₄), and subjected to five rounds of purification (109, 110).

Phage-resistant mutants were selected by infecting log-phase ZZ006 (10^8^ CFU/mL) with VS1 (10^8^ PFU/mL; MOI = 0.1). Surviving bacteria were isolated by streak plating and validated by spot assays and 16S rRNA sequencing (111, 112). Resistant mutant ZZ006M1 served as host for isolating VS2, and subsequently, ZZ006M2 (resistant to VS2) was used to isolate VS3. All strains and phages were stored at −80°C in 25% glycerol.

### Preparation of high-titer phage suspensions

Log-phase cultures (5 mL) were infected with phage lysate (1 mL, MOI = 0.1) and incubated at 28°C with shaking (180 rpm). Upon complete lysis, cultures were filter-sterilized (0.22 µm) and used to inoculate 50 mL of fresh log-phase culture. Following lysis, cultures were clarified by centrifugation (8,000 × *g*, 10 min), filter-sterilized, concentrated using 30-kDa ultrafiltration membranes (UFC5030), and stored at 4°C.

### Observation of phage morphology by transmission electron microscopy

Concentrated phage suspensions were washed three times with sterile ultrapure water using 30-kDa centrifugal filters. Purified phages were applied to Formvar-coated 400-mesh copper grids and negatively stained with 2% (wt/vol) uranyl acetate for 3 min. Samples were examined using a Hitachi H-7650 transmission electron microscope at 80 kV (113, 114).

### One-step growth curve analysis

Log-phase cultures (1 mL) were infected with phage at MOI = 0.01. After 20 min adsorption at 28°C, mixtures were centrifuged five times (6,000 × *g*, 5 min, 4°C) to remove unadsorbed phages. Pellets were resuspended in 1 mL fresh medium, diluted 1:1,000 into pre-warmed 2216E medium, and incubated at 28°C with shaking (180 rpm). Samples were collected every 10 min and titered by double-layer plaque assay (56, 111).

### Phage stability assays

The stability of the phages VS1, VS2, and VS3 under various pH and thermal conditions was assessed according to the methods described by Verma et al. (58, 115, 116) with some modifications. pH stability was assessed by incubating phages (10^8^ PFU/mL) in 2216E medium adjusted to pH 1–12 for 1 h at 28°C. Samples were neutralized to pH 7.6 and titered by plaque assay. For thermal stability, phages (10^8^ PFU/mL) were incubated at 4°C, 20°C, 30°C, 40°C, 50°C, 60°C, and 70°C for 30 days, with titers determined on days 1, 5, 10, 15, 20, 25, and 30. All assays were performed in triplicate.

Chloroform sensitivity was tested by mixing phage suspensions with chloroform at final concentrations of 0%, 2%, and 20% (vol/vol), vortexing vigorously for 1 min, incubating for 30 min, and centrifuging (6,000 × *g*, 5 min). Supernatants were spotted onto bacterial lawns to assess infectivity (117, 118).

### Host range determination of phages and phage cocktails

Spot tests were performed on 24 *Vibrio* spp. and 2 *Shewanella* spp. strains from our laboratory collection (Table 2). All strains were cultured in 2216E broth at 28°C until they reached the log phase (OD_600_ = 0.4–0.6). A 1 mL aliquot of the log-phase bacterial culture was mixed with 6 mL molten 0.5% 2216E soft agar and overlaid onto 1.5% agar plates. Phage lysates (5 µL, 10^8^ PFU/mL) or phage cocktails (VS1 + VS2 or VS1 + VS2 + VS3) were spotted onto the agar surface and incubated at 28°C for 12 h. Clear zones around the spots indicated phage susceptibility (119). All strains grew well on 2216E medium under these conditions, and no growth issues were observed during the incubation period.

### Bacterial growth reduction assay

Log-phase cultures (10^8^ CFU/mL) were co-incubated with phages at MOI 0.1 or 1.0 in 48-well plates. OD_600_ was monitored hourly using a microplate reader (Infinite M200 Pro, Tecan). Viable bacteria were enumerated at 48 h by plate counting. Treatments included single phage (VS1) or cocktails (VS1 + VS2, VS1 + VS3, or VS1 + VS2 + VS3). Bacterial reduction was calculated relative to phage-free controls. All experiments were performed in triplicate.

### Phage genome sequencing and analysis

Phage genomic DNA was extracted using the FastPure Viral DNA/RNA Mini Kit Pro (Vazyme). Whole-genome sequencing was performed on the Illumina platform (OE Biotech, Shanghai, China). Reads were assembled using metaSPAdes v3.15.4 (120) with default parameters.

ORFs were predicted using GeneMarkS (http://exon.gatech.edu/GeneMark/genemarks.cgi) with phage-specific settings (121). Translated proteins were annotated by BLASTP against the NCBI nr database (*E*-value < 10^−5^) (122). Genomes were screened for virulence factors (VFDB) (http://www.mgc.ac.cn/cgi-bin/VFs/blast/blast.cgi), antibiotic resistance genes (CARD) (https://card.mcmaster.ca/home), and tRNAs (tRNAscan-SE 2.0) (http://lowelab.ucsc.edu/tRNAscan-SE) (123–125). Viral phylogenetic trees and genome synteny were analyzed using ViPTree (http://www.genome.jp/viptree) (126).

### Whole-genome resequencing and variant analysis of ZZ006M3

Phage cocktail-resistant mutants (ZZ006M3) were obtained by co-culturing *V. sinaloensis* ZZ006 with the three-phage cocktail. Six independent resistant isolates were selected and propagated for whole-genome resequencing, and their ability to grow in the presence of the phage cocktail was confirmed prior to sequencing. Genomic DNA was extracted using the SDS-column purification method. DNA quality and integrity were assessed by agarose gel electrophoresis and quantified using a Qubit 3.0 Fluorometer (Life Technologies, Carlsbad, CA, USA). Sequencing libraries were prepared using the Ligation Sequencing Kit (SQK-LSK109) and Native Barcoding (EXP-NBD104/EXP-NBD114) for PromethION sequencer (Oxford Nanopore Technology, Oxford, UK) according to the manufacturer’s instructions, and paired-end sequencing (2 × 150 bp) was performed on an Illumina NovaSeq platform (Shanghai Ouyi Biomedical Technology Co., Ltd., China). Raw sequencing reads were processed using Trimmomatic v0.39 to remove adapter sequences and low-quality bases. The resulting high-quality reads were used for downstream variant analysis. Cleaned reads were mapped to the reference genome of *V. sinaloensis* ZZ006, with chromosome I and chromosome II analyzed separately, using Bowtie2 v2.5.1 (127). Alignment files were processed with SAMtools v1.6 (128), and variants were called using BCFtools v1.16 (129). Variants were filtered based on a minimum base quality score of 20 and a minimum read depth of 10. Functional annotation of variants was performed using SnpEff v5.2 (130) with a custom database constructed from the ZZ006 genome and corresponding GFF3 annotation files. Annotated variant files were further processed to extract mutation types (e.g., missense, frameshift, nonsense, and in-frame deletion) and their predicted functional impacts (high or moderate). The resulting variant data sets were used for comparative genomic analysis and identification of mutations associated with phage resistance.

### Statistical analysis

Each experiment was repeated with at least three biological replicates. The obtained data were then presented as the mean with standard deviation (SD).

## Data Availability

Complete genome sequences of *V. sinaloensis* ZZ006 chromosomes 1 and 2 have been deposited in NCBI GenBank under accession numbers CM187676.1 and CM187677.1, respectively. Raw data of the whole-genome resequencing of six independent resistant isolates of three-phage-resistant mutants (ZZ006M3) have been deposited in NCBI GenBank under accession number PRJNA1444768. Complete genome sequences of phage VS1, VS2, and VS3 have been deposited in NCBI GenBank under accession numbers PX251737, PX251738, and PX251739, respectively.

## SUPPLEMENTAL MATERIAL

Supplemental material aem.00680-26-s0001.xlsx Tables S1 to S9.

ASM does not own the copyrights to Supplemental Material that may be linked to, or accessed through, an article. The authors have granted ASM a non-exclusive, world-wide license to publish the Supplemental Material files. Please contact the corresponding author directly for reuse.
